# Empowered Stakeholders: Female University Students’ Leadership During the COVID-19-Triggered On-campus Evictions in Canada and the United States

**DOI:** 10.1007/s13753-021-00362-6

**Published:** 2021-06-18

**Authors:** Haorui Wu, Marla Perez-Lugo, Cecilio Ortiz Garcia, Frances Gonzalez Crespo, Adriana Castillo

**Affiliations:** 1 Canada Research Chair in Resilience, School of Social Work, Dalhousie University, Halifax, NS B3H 4R2 Canada; 2 Natural Hazards Center, University of Colorado Boulder, Boulder, CO 80309 USA; 3 Department of Social Sciences, University of Puerto Rico Mayagüez, Mayagüez, PR 00682 USA; 4 The RISE Network, Mayagüez, PR 00680 USA

**Keywords:** Canada, COVID-19, Female leadership, Gender and disaster, On-campus evictions, United States, University students

## Abstract

The study of disaster-specific leadership of female university students has been largely neglected, especially during on-campus emergency eviction and evacuation. Based on the COVID-19-triggered, on-campus evictions across Canada and the United States, this cross-national partnership examined the out-of-province/state and international female university students’ leadership during the entire eviction process. Through in-depth interviews, this study revealed the female university students’ leadership behaviors during three stages: (1) pre-eviction: their self-preparedness formed an emotional foundation to support others; (2) peri-eviction: their attitude and leadership behavior enabled them to facilitate (psychologically and physically) their peers’ eviction process; and (3) post-eviction: they continued to support their peers virtually and raised the general public’s awareness regarding the plight of vulnerable and marginalized populations. This article argues that the female university students’ leadership that emerged during the eviction process became complementary to and even augmented the universities’ official efforts and beyond. This leadership represents empirical evidence that contributes to the existing literature on gender and leadership by demonstrating female youth as empowered stakeholders rather than as merely passive victims. Future studies could develop detailed stratification of gender and age dimensions in order to portray a more comprehensive picture of the younger generation’s leadership in hazards and disaster research and practice.

## Introduction

Sociodemographic variables (such as gender, age, and ethnic origin) in combination with other societal determinants (including cultural, economic, social, and political) continually shape how people cope with global climate change, disasters, and other world-wide crises (for example, pandemics and terrorism) (Laska and Morrow 2006; Mercer et al. 2010; Goltz and Mileti 2011; Phillips et al. 2012; Peek et al. 2020). In reference to gender, although researchers have been continually promoting gender stratification in hazards and disaster research and practice (Fothergill 1996; Enarson and Fordham 2001; Erickson and Ritter 2001; Jenkins and Phillips 2008), gender-informed studies have begun only relatively recently to focus on women’s leadership (Hou and Wu 2020) rather than to merely examine women stereotypically as passive victims. Children and youth-focused hazard and disaster studies have followed a similar trajectory and have begun to move beyond viewing younger generations as solely vulnerable and marginalized victims (Fothergill and Peek 2015). Cumiskey et al. (2015) illustrated that engaging young people in various disaster-related efforts contributes to their prospective leadership in climate change adaptation and disaster risk reduction. In the context of tertiary education, merging the sociodemographic factors of gender and age highlights a unique group of female university/college students that includes adolescents (17−19 years), youths (20−24 years), and young adults (25−35 years) (Furlong 2013). Existing research has rarely examined these female students’ contributions and leadership in disaster situations (Gomez 2013), especially their involvement in on-campus emergency response (for example, eviction and evacuation).

The global COVID-19 pandemic has generated an opportunity to explore the active involvement of female university students in on-campus emergency response. Intending to prevent community-based coronavirus transmission, starting in March 2020, world-wide shutdowns resulted in university and college campuses being temporarily closed, which triggered widespread evictions of students who were living on campus (Teotonio 2020). These evictions brought on enormous challenges for the in-province/state students in Canada and the United States, and even more so, for the out-of-province/state and international students. Since the out-of-province/state and international students might not have strong local support, they were confronted with the special challenges of having to deal with the emergency evictions on their own.

In April 2020, scholars from Canada and the United States established a cross-national research partnership to qualitatively examine various impacts of the evictions on out-of-province/state and international university students who were living on campuses in both countries. This pilot study reported on emerging themes of female students’ leadership during the entire eviction process. The identification of their leadership behaviors contributed to the existing literature regarding gender and leadership in disaster settings and enabled these students, their peers, and hopefully their universities to better prepare for future potential pandemics and other extreme events.

## Female University/College Students and Disaster-Specific Leadership

Leadership within the scope of disaster and emergency management is vital for building community resilience and sustainability (Trainor and Velotti 2013). This section reviews the literature with respect to the development of disaster and emergency management within the tertiary education environment, including the significance of gender, age, and leadership, the three theoretical pillars that ground this article.

### Top-Down and Bottom-Up Disaster and Emergency Management Leadership in the Context of Tertiary Education

Disaster and emergency management leadership has been discussed at both organizational and grassroots levels. At the organizational level, the main focus has been on professional disaster and emergency management organizations, such as the U.S. Federal Emergency Management Agency (Karaca et al. 2013), the United Nations Office for Disaster Risk Reduction (UNISDR 2015), and other community-based agencies. This organizational leadership aims to coordinate various resources and improve policy and decision making domestically and internationally. Since disaster-related efforts are designed for local disaster survivors and these survivors are the real beneficiaries of disaster-related endeavors, a nascent field of grassroots leadership that took place throughout a full disaster cycle was investigated (Hou and Wu 2020). The grassroots leadership studied mainly concentrates on empowering and broadening the local residents’ diverse expertise to develop and strengthen resilience capacity at individual, family, and community levels (Arnold 2013).

The current COVID-19 pandemic has increasingly directed attention towards leadership within those institutions/organizations that include a high percentage of vulnerable populations, including schools (Harris and Jones 2020), hospitals (Allameh et al. 2020), and long-term care facilities (Brown 2020). In the context of post-secondary education, leadership usually is administered through a top-down approach, starting with the professional emergency managers, and moving down to the second-tier leaders, that is those who directly engage with students, faculty, and staff. This top-down emergency response strategy helps to prevent the loss of lives and reduce the short- and long-term physical, social, and economic impacts of disasters (for example, property damage, the elimination of educational programs and faculty positions, and the suspension and even the discontinuation of some critical ongoing research processes) (Ahmad 2007; CUNY 2021). Hence, decreasing these negative impacts could lead to the examination of the effectiveness of the organizational top-down disaster and emergency leadership arrangement.

Vulnerable and marginalized populations have been disproportionately affected by extreme events; the grassroots leadership, featured in bottom-up empowerment, highlights the various vulnerabilities of these underpresented groups, including women (UNISDR 2015), children and youth (Forbes-Genade and van Niekerk 2019), the elderly (Wu 2020), immigrants and refugees (Carlton 2015), and Indigenous communities (Mercer et al. 2010). Female university/college students feature a dual demographic-specific vulnerability associated with both gender and age (Holbein and Hillygus 2020). Furthermore, the out-of-province/state and international students are at even more risk of vulnerabilities than their peers due to lack of direct family support and local ties. All these vulnerabilities have, in the past and recently, pointed research toward observing young people as a vulnerable, passive, and dependent group, rather than addressing the diversity within this group, and addressing their strengths and leadership capabilities as well (Tanner and Doberstein 2015; Osofsky et al. 2018).

Although building disaster-specific leadership in the post-secondary education context requires the integration of both top-down and bottom-up strategies, specific research remains inadequate, especially with respect to students. University/college students, who have significantly engaged in post-disaster response, are situated in the life stage of keenly absorbing knowledge, learning skills, and obtaining cumulative experience (Nissen et al. 2021). Building their disaster and emergency-related leadership not only benefits their individual development, but also creates a bottom-up approach that strengthens on-campus communities’ disaster and emergency response capacity. This disaster-driven, student-specific leadership will, ultimately, characterize those who educate the next generation of young leaders to serve broader communities, and in the long run, enhance these communities’ resilience and achieve holistic, sustainable development.

### Female Adolescent and Youth Leadership in Disaster Initiatives

The general public’s leadership associated with emergency response actions has been considered as one type of citizens’ “rights and obligations” (Johannisson and Olaison 2007, p. 72). In general, survivors, take on emergency response interventions, have been described as complementary, narrowing the gaps, and/or addressing the failures of official efforts (Majchrzak et al. 2007). Although the mainstreaming culture of disaster and emergency management leadership has been male-dominated (Wilson 1999), an increasing number of female leaders has been shifting this culture towards a more gender-equal perspective (Drolet et al. 2015). Female political leaders and medical officers world-wide have demonstrated their strong leadership in response to the COVID-19 pandemic (Funk 2020). Cvetkovic et al. (2018) indicated that women’s traditional and natural caring characteristics enable them, when disaster hits, to take up leadership roles in support of their families’ safety, health, and well-being, and in helping their neighbors and other people affected by the disaster.

Recent studies have been comprehensively exploring young people’s engagement in the full disaster lifecycle of a variety of extreme events, including pandemics, hurricanes, explosions, and shootings (Peek et al. 2016). These studies have examined various vulnerabilities associated with young people, with the aim of strengthening their resilience capacities (Haynes and Tanner 2015) and improving their physical health, mental wellness, and overall well-being (Slone et al. 2013; Höfler 2014). The catastrophic influences of extreme events have motivated the younger generation’s leadership advocacy for environmental and social justice (Marris 2019). It is vital to design empirical approaches and collaborative networks to navigate different resources, and develop evidence-based strategies that motivate the younger generation’s leadership behaviors. This would not only help prepare the younger generation themselves, but also their families, communities, and societies for prospective extreme events (Ronan and Johnston 2005; UNICEF 2013).

The study of adolescent and youth leadership manifested in various disaster-related efforts has been identified through two major approaches: (1) by examining how youth leadership is developed through community participation and related training programs (Selby et al. 2020); and (2) by examining how extreme events give incentive to these groups to take on leadership (Nissen et al. 2021). Most existing endeavors follow the first path, by engaging young people in global agendas and local initiatives of climate change adaptation and disaster reduction, with the aim of creating effects, starting at the individual level, which then ripple through family, community, and eventually the le of society (Kerr et al. 2018). For example, when adolescents and youth were engaged in post-hurricane Katrina efforts in Louisiana, it enhanced their resilience-based leadership and advanced their “perceived ability to achieve goals fostering post-disaster resilience” (Osofsky et al. 2018, p.11). Gender-specific leadership programs have also been investigated. The South African Girls in Risk Reduction Leadership program concentrated on integrating adolescent girls into community-based decision-making processes, building their leadership in disaster risk reduction (Forbes-Genade and van Niekerk 2019). This second stream that relates to how extreme events stimulate young people to take on leadership has not gained enough attention.

University/college students’ leadership in disaster settings has been briefly discussed. Carlton and Mills (2017) discovered that, after the Canterbury Earthquakes in New Zealand (2010−2011), the non-hierarchical organizational structure of community-based, non-profit disaster response agencies stimulated the leadership of their members, namely university student volunteers, to utilize their strengths and judgement in providing timely emergency assistance. However, most case studies relating to university/college students’ leadership have not elaborated on the influence of the gender factor. In recent years, university/college campus-based pre-disaster preparedness has been drawing the interest of scholars (Tkachuck 2016). Tanner and Doberstein (2015) discovered that students from the University of Waterloo (Canada) were not equipped with the independent capacity to cope with the first 72 hours of extreme events, raising the awareness of the need to improve the university’s administrative support. Since most university students were still treated as passive victims rather than empowered stakeholders, their potential leadership was largely ignored.

The lack of the identification of gender-specific and disaster-driven leadership among university/college students in emergency evictions presents a significant gap in hazards and disaster research and practice. Research on the COVID-19-triggered, on-campus evictions provides a valuable opportunity to address this research deficit. In focusing on out-of-province/state and international university/college students from Canada and the United States, this pilot study aimed to develop a more profound understanding of these students’ challenges and solutions during the entire eviction process, in order to provide evidence-based strategies for the universities to improve their emergency response endeavors for potential extreme events. Gender and leadership are the two emerging dimensions from this study that directly contribute to this mission.

## Research Design

In response to collecting time-sensitive data regarding the varying societal impacts of eviction on out-of-province/state and international university students living in on-campus housing, this study employed the qualitative data collection instrument of the in-depth interview method to explore these students’ eviction experiences. In order to facilitate this cross-national partnership, the research ethics application was approved by the two countries’ investigators’ primary affiliations, that is by the Research Ethics Board at Dalhousie University, Halifax, Canada, and the Institutional Review Board at the University of Puerto Rico Mayagüez, the United States.

### Research Settings and Participants

During May and June 2020, both convenience and snowball sampling approaches (for example, online advertising and sending invitations through students’ service email lists) were utilized, aiming to approach potential participants as swiftly and effectively as possible and to collect time-sensitive data (Norris 2006). This pilot study invited 20 out-of-province/state and international students attending both Canadian and American universities (10 participants from each country, respectively) to participate in in-depth individual on-line interviews (45−60 minutes) (Table 1). Among them, 55% were female students (six in Canada and five in the United States). Since 12 interviews are generally considered sufficient to achieve theoretical saturation in qualitative research (Guest et al. 2006), this sample size of 20 participants features a reasonable data curation foundation for a homogeneous group of university students (Green and Thorogood 2018; Dworkin 2012). In each country, students were enrolled in graduate and undergraduate programs in research-intensive universities, reflecting the nature of the post-secondary education system of each country (for example, all the Canadian universities that the participants attended are public universities, while the U.S. universities are a mix of private and public universities). The geographic locations of these universities are spread across each country. The interview questions were designed to identify the students’ eviction challenges and solutions, at both individual (students’ bottom-up approach) and organizational (universities’ top-down approach) levels. This two-way data curation encourages the interpretive lens to analyze the interplay between students’ responses and their universities’ official efforts.

**Table 1:** Participants’ demographic composition in the study on female university students’ leadership during the COVID-19-triggered on-campus evictions in Canada and the United States

		Canada (10 participants)	U.S. (10 participants)
Gender	Female Male	6 (60%) 4 (40%)	5 (50%) 5 (50%)
Programs	Undergraduate Graduate	5 (20%) 5 (50%)	9 (90%) 1 (10%)
Immigration status	Out-of-province/state International	5 (50%) 5 (50%)	9 (90%) 1 (10%)
Ethnic Groups	White Hispanic and Latino African Decent Asian Mixed	4 (40%) – 3 (30%) 2 (20%) 1 (10%)	4 (40%) 6 (60%) – – –

### Data Analyses

**Fig. 1:**
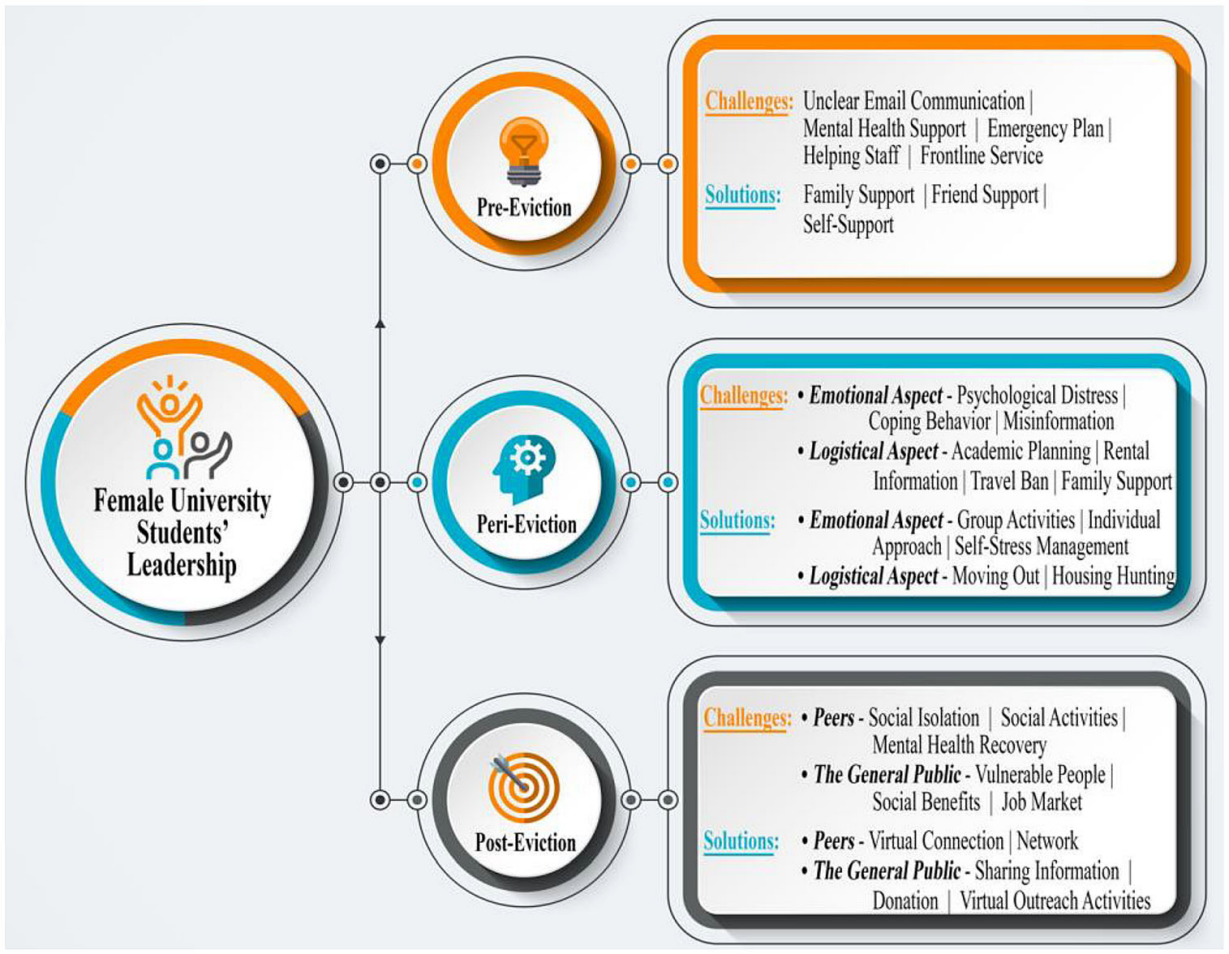
Themes (circles) and codes (boxes) were generated in the analysis of female university students’ leadership during the COVID-19-triggered on-campus evictions in Canada and the United States

The 20 interviews were audio-recorded (verbal consents were obtained at the beginning of the audio-recordings), transcribed verbatim, and analyzed using a qualitative thematic approach through two stages of data analyses (Norris 2006; Saldaña 2009). The first stage of data analysis followed the interview questioning sequence by identifying the students’ various reactions during the entire eviction process, generating the themes related to the challenges that the students confronted and the solutions they developed. The gender-specific themes of female adolescent and youth leadership emerged during this first stage. The second stage of data analysis integrated the eviction phase dimension (pre-, peri-, and post-eviction), in order to assess these students’ varying degrees of engagement during each stage. This stage of the data analysis further identified the categorization of the students’ efforts, visualized their entire emergency preparedness and response, and identified the promising practices that official eviction efforts could consider. The final codes and themes used for the analysis are shown in Fig. 1 (the circles show themes and sub-themes and the boxes present codes).

Both stages of data analysis uncovered that the participants in both countries shared tremendous similarities regarding their eviction experiences and also presented differences. The differences were rooted in various societal factors (for instance, social, cultural, and political) of Canada and the United States, and both countries’ differing emergency response policies at federal, province/state, and municipal levels. These differences did not significantly emerge among the themes regarding the female students’ leadership. Hence, variation of country is not considered to be a major determinant. Furthermore, although the nine male student participants’ narratives illustrated some leadership relevant behaviors during the eviction (for instance, facilitating the move-out process and helping search off-campus housing information), their leadership had not continually emerged during the overall data analysis process. Synthesizing the themes from the two stages of data analysis contributed to the conceptualization of the female students’ leadership during the different stages of eviction, which is detailed in the next section.

## Female University Students’ Leadership Pre-, Peri-, and Post-Eviction

The themes related to female students’ leadership chronologically manifested in the three major stages of the eviction, that is pre-, peri-, and post-eviction. During each stage, sub-themes were specified to demonstrate the leadership contributions made on behalf of themselves, their peers, and beyond. The sub-themes are primarily supported by the 11 female student participants’ narratives. The nine male student participants’ narratives served as peers’ observations to reflect and confirm the female students’ leadership.

### Pre-Eviction: Uncertainty and Emotional Readiness

The swift unfolding of COVID-19 generated tremendous unpredictability for the entire world (Cash and Patel 2020). These uncertainties were reflected in universities’ emergency response plans across Canada and the United States, especially in the eviction-related risk communications that were circulated by the university authorities.

Consequently, all the participants identified two common issues in their universities’ emergency response strategies and risk communications. (1) The top-down risk communications by emails were unclear, inconsistent, and not transparent. One male participant provided an example, showing that the university used vague and contradictory language in emails sent out, such as “you may leave” and “you may stay” in one email and “the deadline for moving out is…” in another email. These emails reflected that the universities did not have fixed emergency response plans. These uncertainties worsened the students’ feelings of uncertainty and fear, and negatively affected their personal eviction planning. (2) The emergency response plans at the university level needed to be improved in order to better facilitate the evictions. One male participant indicated that: “I feel that my university did not know how [to facilitate evictions] either. [The university] sent daily emails only. No one came to help us. We all figured out everything by ourselves.” Since the students were accustomed to following their universities’ instructions, these two major issues caused chaos for the students who were living on the campuses. Two female participants indicated their concerns:

Usually, I just do whatever the university tells me to do. [But this time], I didn’t really know if I had a specific way of coping with everything because there were no instructions given in the emails.

I contacted the student housing office and was told that “we received the same email as you!” Why am I gonna keep asking them for information that they don’t have? I would [rather] look into other sources online to see what other universities were doing.

“We haven’t faced something like this [COVID-19] in the majority of people’s lifetimes,” one male participant emphasized. Upon receiving the eviction order, it is understandable that out-of-province/state and international students were initially tempted to connect with their families because they might not have had enough local ties. Some female students described how they emotionally prepared for this emergency, which created the foundation for their various leadership behaviors in the eviction process that followed. One female participant (international freshman) utilized a self-suggestion approach to put herself in a good mood: “You have to be independent. Everyone is in the same boat and they might have more challenges than you that you don’t know about. Taking care of yourself is helping others.” A social work undergraduate student (out-of-province/state), who graduated in May 2020 (approximately two months after the eviction notice was released), indicated that her mother helped her emotionally prepare for eviction: “My mother encouraged me: ‘You have almost graduated. Now you will work as a social worker to help others! You can do it.’”

### Peri-Eviction: Facilitating Self-Eviction and Supporting Their Peers’ Eviction

“Where to move? Where to find related information? How to move?” Students asked these questions to guide their eviction. When most participants recalled their overall eviction experience, they strongly felt that the moving out agenda could have been more easily addressed if their mental wellness had been better supported: “Everyone was overwhelmed when it came to the end of semester. Eviction brought more [stress]. I think mental support should have been available [along with the eviction order].” “The whole campus was in chaos; I did not think [the university’s mental health services] were available.” These concerns motivated some female participants to emotionally prepare themselves to facilitate the entire process of their own and their peers’ eviction.

#### Emotional Support

The misinformation regarding COVID-19 that was spread by social media caused a significant emotional burden for the general public (Islam et al. 2020), especially for young people, such as university/college students (Bengtsson and Johansson 2020). The possible reasons may be that these young people are not yet well equipped with effective enough judgement capacity to screen and verify the authenticity of social media information, nor to be able to positively and correctly interpret that information. Moreover, in March 2020, an increasing number of countries imposed restrictions on international travelers through their public health protocols, only allowing essential personnel to travel (Nardi 2020). This made the out-of-province/state and international students extremely worried about their upcoming travel plans, immediately following the academic semester in April. All these factors collectively increased the students’ mental stress. There was even a rise in unfavorable coping-motivated behaviors (for example, destroying the university properties and spreading bad rumors) among on-campus resident students (Sharma et al. 2020).

A female participant (out-of-province/state undergraduate student) said “I could not control other people [on Facebook]. I just tried to close my eyes to all the news!” The compounding influences of misinformation and the stress of the evictions motivated some female participants’ emergent leadership actions to emotionally support their peers at both group and individual levels.

*Group Interventions*: The following two examples of group interventions showed these female participants’ emergent leadership that helped to bring about emotional stability to their peers during the challenging situation. One female participant in her junior year (out-of-province/state student) shared:

When I heard they were talking about the emails and news in the sitting room, I would jump in and say, “Hey, let’s forget this for a while and have some fun. I found a great movie on Netflix, let’s have some ice-cream and watch it together.” I know everyone was worried about the eviction and a little break was really helpful. I also played card games with them to distract them.

One female participant (first year out-of-province/state graduate student) carpooled with two of her roommates (two freshmen, male) to drive them approximately 14 hours, to get them back home. They chose to drive to avoid the infection risk of taking public transport, which would have been a threat to themselves and their families. The two freshmen were very afraid about the long drive. The girl bravely influenced the two freshmen by emphasizing: “We have a plan! No worries! It’s just a 14-hour trip, everything will be fine.” Then this female participant confided, “Actually, I was more scared than them. I have never had such a long drive. If I showed them my worries, they would be more scared. That does not help at all.”

*Individual Interventions*: At the individual level, the female participants’ leadership pursued individual-specific mental health strategies to address their peers’ various needs. On the night when the university released the eviction order, a female student, who had chronic mental issues, suddenly began to throw her belongings around her room. A female participant (international first-year student) in the psychology master’s program, along with her other roommates, calmed this girl down:

I tried to talk with her first, but it’s not easy at all. You could clearly hear that other students were roaring and complaining very loud outside, which influenced her a lot. I just asked her to listen to me and convinced her that we could get through this together. When she went back to normal, I also requested other roommates to keep an eye on her.

A female participant (out-of-province/state undergraduate student) described how she helped her roommate, who is an international student from Europe:

My roommate just called me and cried on the phone, saying “They evicted us. I do not know where to go!” I replied immediately, “I do not know where to go either but we will figure it out. Take a deep breath in and out… The dining hall will close. Let’s get some groceries first.” Just some very simple things I said, but it worked very well.

#### Relocation “Logistical” Support

When the resident students’ mental conditions seemed to somewhat stabilize, the female participants’ leadership addressed supporting other relocation logistics. The out-of-province/state students could drive or even fly back home. The feasible solution for international students or other domestic students, who were nearing graduation and needed to complete their degree-related activities (for example, internship and field education), was to find off-campus accommodation. Accordingly, searching on-line rental advertisements was the most common strategy. Most on-campus resident students, especially the out-of-province/state and international students, who were in the first two years of their programs, however, might not have had off-campus housing hunting experience. The public health protocols also caused landlords to be apprehensive in accepting new tenants. One female participant (undergraduate student), who worked as a resident assistant (RA) in a student residential hall described the situation:

I began to search and collect any off-campus accommodation information I could find. I called the landlords to confirm [vacancies]. You know some advertisements were no longer available and also some landlords might change their minds [due to COVID-19]. I also sent this list to other RAs.

During the eviction process, some university staff needed to deal with emergency issues and were not always available. This RA explained her role as a frontline worker to support other students in dealing with their evictions: “I tried my best to answer their questions. My supervisor was not always available; I had to make some urgent decisions by myself.”

Finding the rental information was the first step. The female participants also accomplished other logistical tasks that were connected to different aspects of the evictions, such as negotiating with car rental companies for carpooling and moving out, purchasing and distributing packing supplies and groceries, assisting the dormitory residents to quickly pack, to move heavy boxes into storage places, and to drive their peers to airports. A female participant (out-of-province/state graduate student), who had completed her bachelor’s degree at another university in the same city, made herself available to consult with her peers about their moving-out and house hunting plans: “I talked with several international students about their moving decisions, like where are the safe areas? What about other resources nearby, like grocery stores, gyms, and bus service? How much is a reasonable rent?”

### Post-Eviction: Virtual Peer Leaders and Responsible Citizens

After the students had moved off campus, the world-wide COVID-19-triggered shutdown quarantined most of the relocated students at home or in their new places. Another way the female participants displayed their leadership was by creating virtual communities to continually support their peers and others. In this way, they fulfilled two purposes—serving as peer leaders to students from their universities, and serving as responsible citizens to the general public.

#### Virtual Peer Leaders, Continually Supporting Their Peers

Some female participants created virtual communities to continually connect with and support their peers. One of the female participants (out-of-province/state undergraduate student) created a weekly check-in to confirm her previous dormitory residents’ safety and extended her social network among her dormmates and others.

I asked them [all my dormmates] to check-in with one another, like once per week. They were just very simple text messages, but you never know what good things might happen…Some of my housemates still lived in town. I connected them with my other friends over there. In case of emergency, they could support each other.

Another female participant (out-of-province/state graduate student) was concerned about the international freshmen students, who were unable to return to their home countries:

I really do not want the pandemic to destroy their freshman memories… I organized a video group chat, almost every week. I wanted to make sure that everyone was OK… I was told that a girl [one of the freshmen] also had a study group going on with those in the same program. Although they were just behind the screen and doing their own thing, this helped them to not feel lonely.

These female participants utilized these virtual technologies to unite their peers and support one another in facing this challenging period together. As one male participant (international graduate student) indicated, “these text messages or Facetime talks were really helpful, supporting me through the shutdown. I always felt very happy after talking with my roommates.” Another male participant (international first-year graduate student) pointed out that he made more virtual friends during the shutdown than he had during his first year at university. The psychological and wellness benefits accomplished by simple text messages or video chats contributed to the evictees’ overall well-being on an ongoing basis. The female participants’ deep concern for their peers’ emotional well-being extended to their social life. Connecting their dormmates with their friends who were living in the same locality, further assisted these peers to extend and establish their social network and social connections, and to support the international freshmen’s university experience. The positive ripple effects benefited more people in the broader communities.

#### Responsible Citizens

The cultural, social, economic, and political impacts of COVID-19 will last much longer than the physical health consequences, especially with respect to the particularly catastrophic impact on vulnerable and marginalized groups (Wu and Karabanow 2020). COVID-19 has physically isolated communities but, in another sense, it also has socially united people to collaboratively advocate for the basic rights of vulnerable and marginalized groups (United Nations Department of Global Communications 2020). Some of the female participants’ leadership even addressed this mission.

As in many other countries, both Canadian and American government agencies offer emergency programs for eligible citizens, such as the Canada Emergency Response Benefit and the U.S. CARES Act (Zimonjic 2020). Canada also released a special stream of assistance for students, the Canada Emergency Student Benefits (Government of Canada 2020). Two female participants (out-of-province/state undergraduate students), who were eligible for these government assistance programs, leveraged these programs to benefit people who were ineligible.

One female participant, who has a chronic illness and recently graduated, was worried about her medical bills because her student insurance had expired upon graduation, and the pandemic had delayed her potential employment.

I was very lucky because I got the emergency benefit. I used it to cover my drug bill and my other costs. I know a lot of people who were not eligible have medical needs or other urgent issues. I donated a part of my benefits to them.

This participant mentioned that one of her classmates voluntarily coordinated a community-based donation of groceries for people of ethnic minorities who lost their jobs. Inspired by that, she created some critical dialogues on social media to attract people’s attention to address vulnerable and marginalized groups. “I know people stay at home and Facebook would be a great place to spread this information. We really need to think about these inequalities, help those in need.” She added: “I wish I could donate more. I wish I had more power to help others! You do not have to donate; there are other things you can do as well.”

Another female participant, who had received government emergency benefits, and had initially planned to find a job in the summer of 2020, to save for her next school year, decided to give up her plan. She explained, “I know my international classmates were not able to get the benefits. Jobs are more urgent for them.” She shared the information regarding how to apply for the government emergency benefits in her peer groups and simultaneously released a request to those who were eligible for these benefits, saying “I asked them to consider leaving jobs to others because there are so many people unemployed and not eligible for the emergency benefits!”

Both of these female participants live with a number of vulnerabilities, including chronic illness and unstable financial status. Their leadership in raising other people’s awareness of the difficulties undergone by vulnerable and marginalized populations clearly conveys a powerful message that the younger generation is taking on their responsibilities as honorable citizens, committed to building a just society.

## Discussion

Based on the leadership behaviors presented above, this section outlines these students’ leadership contributions towards university-based emergency response strategies and disaster-specific gender issues in literature and practice.

### Leadership and Failures of Frontline Services

The COVID-19 pandemic not only promoted a thorough examination of national public health systems, but also stimulated the call for the imperative improvement of community-based emergency response plans (Guttry 2020). Although an increasing number of universities have already started acting on this urgent area of emergency response, especially related to their overall administration (Tanner and Doberstein 2015), the female participants’ leadership activities pre-, peri-, and post-eviction further exposed the organizational weaknesses in frontline services. The female participants’ leadership was elicited by COVID-19—specifically, they were closely linked with the uncertainties of the pandemic. These uncertainties initiated the universities’ emergency response to close campuses to prevent community transmission. However, the universities’ failures in providing detailed instructions and related support and services, bound up with the stress of the evictions, generated additional challenges and even more burdens for the students. These uncertainties in emergency situations, and the areas in need of improvement in emergency services, inspired the female students’ leadership in preparing themselves for their self-eviction, and kindled the spirit with which they mentally and physically facilitated others’ eviction process. The evidence powerfully contributes to the existing literature by demonstrating that female students, or university students, are not mere passive victims (Tkachuck 2016) but also empowered stakeholders. This contribution will switch the academic focus from only examining these groups’ vulnerabilities to studying the empowerment of their leadership, to further support resilience during diverse disaster responses.

Applying an interpretive lens to critically examine these female participants’ leadership attitudes and activities identifies different areas for improvement within universities’ emergency administrative systems. Before the eviction, self-suggestion and family support emotionally primed these female participants to independently prepare for and cope with this emergency. This preparedness enabled these students to apply similar strategies to emotionally comfort and support their peers and to address their unique requirements during the eviction process. The preparedness further encouraged these participants to continue to pursue leadership in providing others with support (for example, providing rental information and driving home together). These emotional and physical efforts, in turn, reinforce the concept that frontline services must be embedded in university emergency response strategies (Rohli et al. 2018).

The leadership behavior of the female participants not only was compatible with the universities’ emergency responses, but more importantly, addressed the shortcomings of these official efforts (Majchrzak et al. 2007). Consequently, guaranteeing frontline services (for example, mental health services and extending emergency staff’s working hours), would smoothly facilitate students’ evictions and should be strengthened in universities’ organizational emergency management plans. Potential research could examine how to increase student engagement in the university emergency decision-making process to improve the official efforts in better serving students and other on-campus stakeholders.

### Unique Leadership as Peer Leaders and Responsible Citizens

Historically, the field of disaster and emergency management has been male-dominated (Enarson and Pease 2016). As mentioned above, although the male participants’ narratives indicated some leadership-related activities during the eviction, the male students’ leadership behaviors were not as outstanding and continually emerged as their female peers. Hence, this study provides a different approach to addressing gender disparities in disaster settings. During the different stages of the eviction process, the leadership that was taken on by female participants illustrated the positive influence it had on themselves, their peers, and the general public. Their leadership serves as a valuable reference in the contribution of literature on gender and gender disparity in disaster and emergency management research and practice (Drolet et al. 2015), and ultimately challenges the existing male-dominated mainstream cultural perspective within disaster and emergency management (Wilson 1999). Their leadership belies the existing argument regarding the younger generation’s vulnerable status in hazards and disaster research. Based on this study, further detailed stratified research regarding gender (for example, female, male, and non-binary) and age (for example, adolescents, youth, and young adults) would portray a more comprehensive picture of university students’ leadership in disaster situations.

The female participants’ leadership behaviors at the group level (for example, discussions about eviction plans, and escorting freshmen to their homes) correlate well with their original caring nature. After safely relocating, these young women’s strategic leadership behaviors, focused on raising public awareness of the inequalities that vulnerable and marginalized populations have been experiencing, shows their good citizenship. All of these variables, such as gender and age, as well as the citizen responsibilities, will boost the development of related educational programs to amplify the benefits of the younger generation’s leadership, during the current pandemic and during future extreme events.

## Conclusion

The COVID-19 pandemic has presented opportunities to redress weaknesses and inequalities at various levels of society and improve the coping capacity during prospective extreme events. Based on the coronavirus-triggered, on-campus evictions across Canada and the United States, our cross-national partnership is contributing to an understudied field of female university students’ leadership, from the perspective of out-of-province/state and international university students. The female students’ attitudes and actions of leadership before, during, and after the evictions not only enabled them to deal with this first-time experience (the pandemic and the subsequent evictions), but also enabled them to facilitate (psychologically and physically) their peers’ eviction processes and move on. After the eviction, these young women remained in leadership roles and continued to give their peers virtual support. They also raised the general public’s awareness of the plight of vulnerable and marginalized populations, who have been disproportionately affected by the pandemic.

This study raises important considerations of wider significance for reconceptualizing the best strategies in disaster and emergency management research, practice, and education, in general, and in strengthening campus-based emergency response plans by raising and considering students’ voices, in particular. Although female university students are still mainly treated as passive victims during most disaster situations, their leadership behaviors during the COVID-19-caused evictions became complementary and even augmented the universities’ official efforts. This pilot study could serve as a catalyst to stimulate other studies with more detailed stratification of gender and age dimensions, in order to contribute to a more comprehensive picture of the younger generation’s leadership in disaster and emergency management.
